# Fibre Distribution Characterization of Ultra-High Performance Fibre-Reinforced Concrete (UHPFRC) Plates Using Magnetic Probes

**DOI:** 10.3390/ma13225064

**Published:** 2020-11-10

**Authors:** Lufan Li, Jun Xia, Chee Chin, Steve Jones

**Affiliations:** 1 Department of Civil Engineering, Xi’an Jiaotong-Liverpool University, Suzhou 215123, China; lufan.li@xjtlu.edu.cn (L.L.); chee.chin@xjtlu.edu.cn (C.C.); 2 Institute for Sustainable Material and Environment, Xi’an Jiaotong-Liverpool University, Suzhou 215123, China; 3 Department of Civil Engineering, University of Liverpool, Liverpool L69 3GH, UK; Stephen.Jones@liverpool.ac.uk

**Keywords:** C-shape magnetic probe test, fibre orientation angle, flexural test, attenuation factor

## Abstract

Ultra-high performance fibre reinforced concrete (UHPFRC) is an innovative cement-based engineering material. The mechanical properties of UHPFRC not only depend on the properties of the concrete matrix and fibres, but also depend on the interaction between these two components. The fibre distribution is affected by many factors and previous researchers had developed different approaches to test the fibre distribution. This research adopted the non-destructive C-shape ferromagnetic probe inductive test and investigated the straight steel fibre distribution of the UHPFRC plate. A simplified characterization equation is introduced with an attenuation factor to consider the different plate thicknesses. The effective testing depth of this probe was tested to be 24 mm. By applying this method, fibre volume content and the fibre orientation angle can be calibrated for the entire plate. The fibre volume content generally fulfilled the design requirement. The fibre orientation angle followed a normal distribution, with a mean value of 45.60°. By testing small flexural specimens cut from the plates, it was found out that the mechanical performance (peak flexural strength) correlates with the product of fibre volume content and cosine fibre orientation angle.

## 1. Introduction

Concrete has been the most significant construction material throughout history. Ultra-high performance concrete (UHPC) was developed as a cementitious composite material with far higher strength, durability, and resistance to external environments than traditional construction materials [1]. With the addition of fibres, the mechanical properties of ultra-high performance fibre reinforced concrete (UHPFRC) can be further improved especially the performance under tensile loading [2]. Moreover, the resistance to impact, chemical degradation, abrasion, and fire can also be improved [3]. UHPFRC has been widely used in airports [4], bridges, roofs, and cladding [5].

Comparing to the ascending branch of the stress-strain curve, the fibre addition always has a more significant impact on the cracking stage of UHPFRC. The fibre acts as a ‘bridge’ to bond the separated cracked concrete matrix and then being pulled out gradually, which is also known as the ‘bridging effect’ [6]. It compensates for the disadvantage of UHPC of being too brittle. However, if the fibres are distributed poorly inside concrete, for example, perpendicular to the loading direction, the bridging effect will be adversely affected.

Many factors can influence the fibre distribution, for example, fibre volume fraction [7,8], external vibration [9], and mixing sequence [10]. Previous researchers have developed various testing methods to test the fibre distribution inside the concrete matrix, for example, image analysis [10,11,12], AC-IS (AC-Impedance Spectroscopy) [13,14,15], X-ray scanning [16,17], and the magnetic core inductive method [18,19]. Compared to these testing methods, the C-shape ferromagnetic probe method is more economical and convenient, so this method is chosen to be the detecting method in this research.

The C-shape ferromagnetic probe test was proposed by Faifer et al. [20]. However, the main focus of their research was on the theoretical background modelling and derivation of the probe, for example, the simulation of flux density of the probe [21]. The application of this probe was also limited to the basic monitoring of fibre volume content and fibre orientation angle of steel fibre reinforced concrete [21]. In 2016, Nunes et al. made a simple and cheap C-shaped ferrite core with coils wound on it [22]. They further applied this testing method on thin UHPFRC plates. By placing the probe on 300 mm × 300 mm × 30 mm specimens in different directions and testing the inductance, the corresponding fibre distribution conditions can be identified. According to Nunes et al., a relative magnetic permeability µ_r,mean_, which reflects fibre content, can be calculated as:

(1) $$\mu_{r,\varphi }=\frac{L_{\varphi }}{L_{\mathrm{air}}}$$

(2) $$\mu_{r,\left(90{}^{\circ}-\varphi \right)}=\frac{L_{\left(90{}^{\circ}-\varphi \right)}}{L_{\mathrm{air}}}$$

(3) $$\mu_{r,\mathrm{mean}}=\frac{\mu_{r,\varphi }+\mu_{r,\left(90{}^{\circ}-\varphi \right)}}{2}$$

where L_φ_, L_(90°−φ)_, and L_air_ represent the magnetic inductance at φ, (90° − φ), and in the air.

The relationship between the orientation indicator ρ_Δ_ and the relative magnetic permeability can be expressed as:

(4) $$\rho_{\Delta }=\rho_{\left(90{}^{\circ}-\varphi \right)}-\rho_{\varphi }=\frac{\mu_{r,\left(90{}^{\circ}-\varphi \right)}-\mu_{r,\varphi }}{2\left(\mu_{r,\mathrm{mean}}-1\right)}$$

Nune et al.’s later research was mainly emphasizing on the detection of uniformly distributed UHPFRC by aligning the fibres with an intense magnetic field [23,24], but the intrinsic heterogeneity of fibre distribution had been proved to largely affect the tensile response of fibre reinforced concrete [25]. Moreover, recent studies were mainly focusing on the relationship between fibre content and flexural performance [26,27]. Therefore, both the local fibre spatial and orientation distribution should be taken into consideration, but this area still needs further investigation.

In previous research, the magnetic probe method was only applied on thin plates. If this method is going to be applied for the purpose of checking the structural safety, it is necessary to determine the effective depth of the probe. In order to fill the gap, the effective depth of the magnetic probe was firstly examined and an attenuation factor was obtained to further correct the fibre volume content data. Apart from this, a simplified analytical solution was derived to determine the relationship between the fibre orientation angle and orientation indicator. In addition, compressive tests and flexural tests had been conducted and the correlated equation between mechanical performance and the tested fibre distributions was derived. The corresponding theoretical explanation of strain-hardening behaviour was clarified.

## 2. Material Preparation and Concrete Mixing

### 2.1. Materials and Specimen List

**Figure 1:**
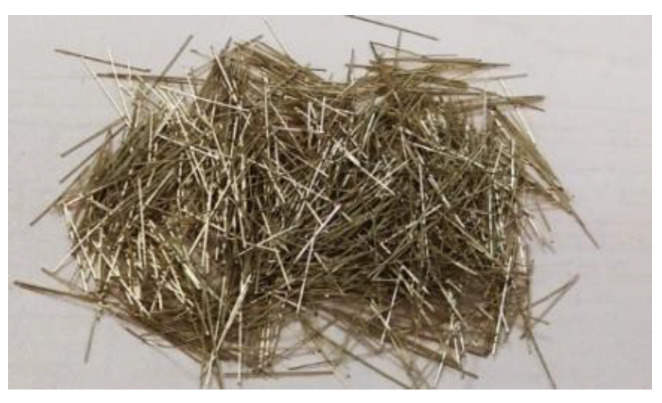
Straight steel fibres used in research.

Premix powder material provided from third party company (Beirong Circular Materials Co., Ltd., Yingtan, Jiangxi, China) were used to ensure the concrete grade in each batch. The premix powders contained the basic components needed for UHPFRC (cement, fine sand, silica fume, and quartz). Straight steel fibres (as shown in Figure 1) covered with a brass coating were added in the mix. The diameter and length of fibres were 0.25 mm and 12.5 mm respectively, given an aspect ratio of 50. The uniaxial tensile strength of the fibres can go up to 2850 MPa. Polycarboxylate superplasticizer was added to ensure the workability. Detailed mix proportions are presented in Table 1.

Table 2 lists the dimensions and casting purpose of all specimens. Cubes were used to determine the compressive strength. Plates with different thicknesses were designed to detect the fibre orientation and spatial distribution, and the following flexural performances.

### 2.2. Concrete Mixing Process and Curing Condition

Table 3 shows the suggested mixing procedure and mixing time provided from the supply company. Considering the differences of the mixing environment and fibre amount, the mixing time varied by ±30 s.

**Figure 2:**
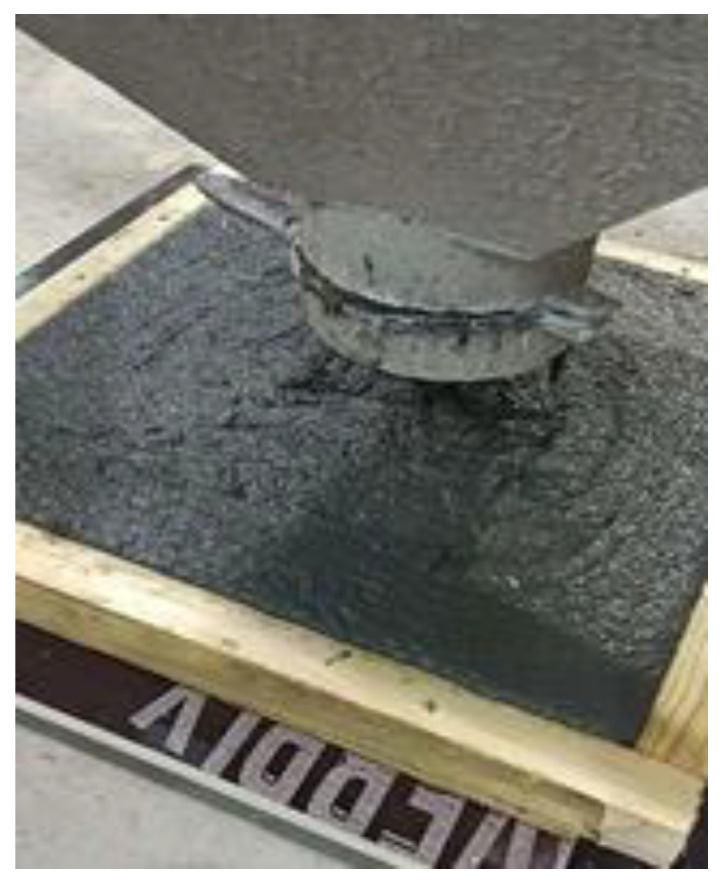
Plate casting direction.

All the cube specimens were cast in three layers with minor hand vibration to expel the air trapped in the fresh concrete. For plates, concrete was poured from the centre and let it flow freely towards the four edges (see Figure 2). To avoid the segregation of fibres, no vibration was applied.

All the fresh concrete specimens were covered with a plastic film to prevent the early shrinkage due to water evaporation. After 24 h of hardening, all specimens were demolded and moved to a steam curing tank. The curing duration included 2 h for increasing the curing temperature to 90 °C and then another 48 h constant curing at 90 °C.

## 3. C-Shape Magnetic Probe Test

### 3.1. Probe Specification

**Figure 3:**
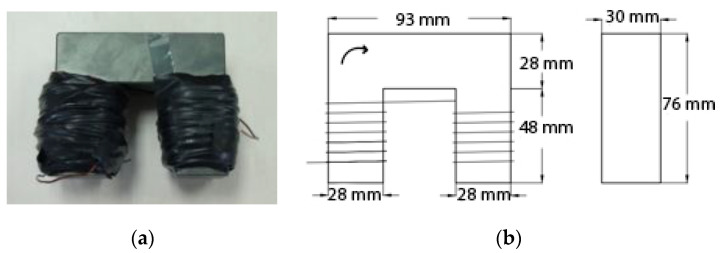
(**a**) Appearance of the magnetic probe and (**b**) detailed dimensions of the ferrite core.

A magnetic probe was manufactured based on Nunes’ research [22]. The probe (Figure 3a) was made of a high frequency inductive Mn-Zn ferrite core wrapped by 350 turns of 0.9 mm diameter enameled copper wire, then tightened by black insulated rubber tape to protect the safety of users. The ferrite core used in this research was 76 mm tall, 93 mm long, and 30 mm wide. Detailed dimensions of the magnetic probe can be seen in Figure 3b.

The inductive test was carried out after curing. The magnetic probe was placed on a smooth surface of the UHPFRC specimen and connected with a LCR meter with two clips. Tonghui TH2830 LCR meter was used to measure the magnetic inductance under 1 kHz with a test signal of 1 V. The variation of inductance of a single object was lower than ±0.01 mH under this testing condition.

### 3.2. Effective Depth Test Method

**Figure 4:**
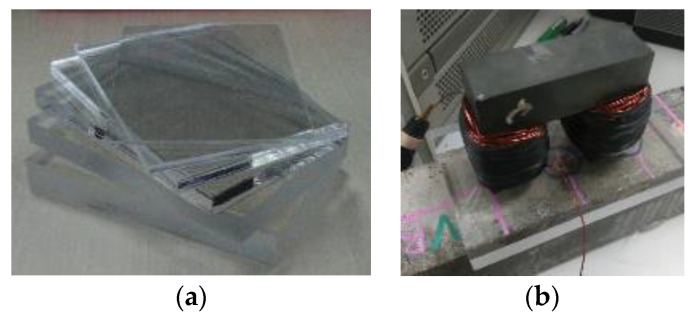
(**a**) Acrylic plates used in the experiment and (**b**) experiment set up of an effective depth test.

Non-ferromagnetic object like wood, plastic, and glass did not show any impact on the inductance data. By placing 100 mm × 150 mm non-ferromagnetic acrylic plates (Figure 4a) with different thicknesses (0, 2, 4, 8, 12, 15, 17, 20, 24, 28, 32, and 36 mm) between the specimens and the magnetic probe (Figure 4b), the magnetic inductance data can be measured and the relative magnetic permeability (RMP) µ can be calculated as:

(5) $$\mu =\frac{L_{0}}{L_{\mathrm{air}}}$$

where

L_0_  magnetic inductance when placing the magnetic probe directly on the specimen;
L_air_  magnetic inductance when placing the magnetic probe in the air and far from any conductive object.

However, since the initial relative magnetic permeability for each group was different, it was difficult to reflect on how the relative magnetic permeability decayed with the increase of thickness. Thus, an attenuation factor (AF) was introduced to describe the residual proportion of magnetic permeability. It can be calculated as:

(6) $${\mathrm{AF}}_{t}=\frac{\mu_{t}-1}{\mu_{0}-1}\times 100\%$$

where

µ_0_  magnetic permeability when placing the probe directly on the specimen;
µ_t_  magnetic permeability at thickness t.

### 3.3. Effective Depth Test Results

The effective depth testing was conducted on 2% and 2.5% vol. UHPFRC. In total, 4 points (2 points for each group) were tested. Based on Equation (6), AF data of each testing point were calculated and the results are shown in Table 4. The relative magnetic permeability decreased with the increase of plate thickness. For specimens with lower fibre content, the relative magnetic permeability tended to drop quicker. All groups of AF data dropped below 10% for depths greater than 24 mm. This proved that the fibres 24 mm away from the testing surface had little effect on the relative magnetic permeability.

**Figure 5:**
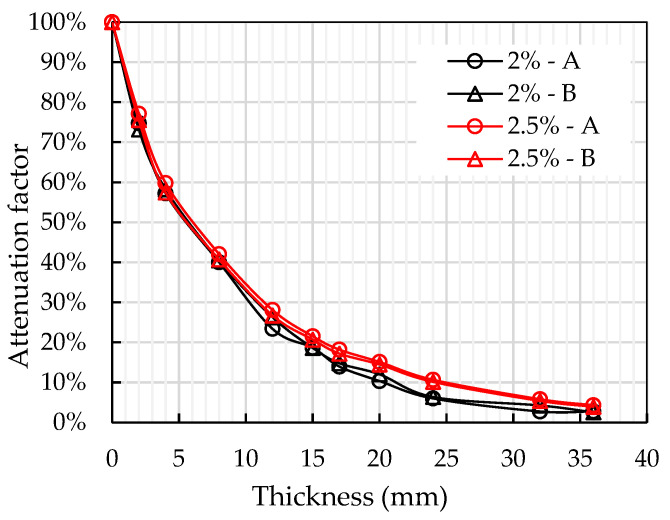
Attenuation factors of 2% and 2.5% vol. specimens at different thicknesses.

The AF data presented in Figure 5 shows very similar exponential trends for all groups of testing points. It proved that the fibres closer to the testing surface had a more significant effect on the relative magnetic permeability, especially within the top 6 mm of the testing surface. After the top 6 mm, the AF dropped below 50%.

To get a theoretical expression of this relationship, a curve fitting analysis was conducted using MATLAB. The relationship between the testing depth and attenuation factor can be expressed by Equation (7) with an R-squared value equal to 0.995.

(7) $$\mathrm{AF}=e^{-0.115\;\times \;t}$$

This test revealed the effective depth when using this particular probe. If any thin specimens are going to be tested in the future, AF can be used for correcting and calculating the real fibre volume content value.

### 3.4. Plate Test Method

**Figure 6:**
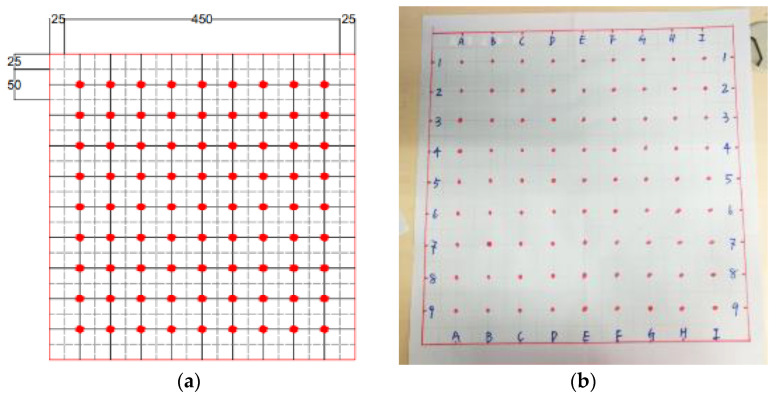
Testing area divisions of the UHPFRC plate. (**a**) Schematic graph (unit: mm) and (**b**) practical experimental design.

The magnetic probe test of all the plates were tested right after finishing curing. The bottom surfaces during casting were used as the testing surface as they were smooth and flat. Considering the length of the magnetic probe was 93 mm, the 500 mm × 500 mm plates were labelled on a 9 × 9 grid from A1 to I9 at equal distances of 50 mm (Figure 6a). A paper testing map (Figure 6b) with 81 points highlighted in red was made to accurately locate the testing points.

**Figure 7:**
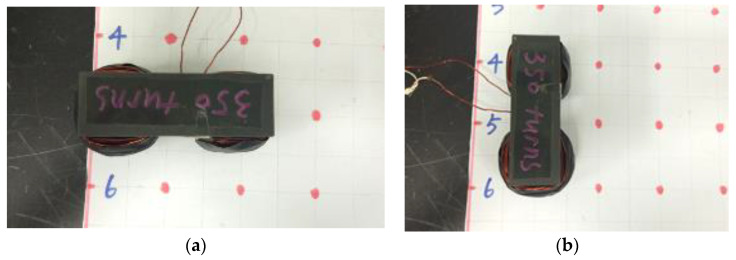
Testing directions. (**a**) Horizontal and (**b**) vertical.

As shown in Figure 7, by placing the magnetic probe in two orthogonal directions (horizontally and vertically), the spatial distribution and orientation distribution at each red point were measured.

The magnetic inductances of the red points in Figure 6 were recorded directly from the LCR meter. In total, 81 data points were collected for each plate. Air inductance was labelled as L_air_. The magnetic inductance values measured in the horizontal and vertical directions were labelled as L_ij,x_ and L_ij,y_. All the magnetic inductance values were divided by the air inductance to get the relative magnetic permeability µ.

The average of relative magnetic permeability measured in two orthogonal directions is the indication of fibre volume content. Based on Equation (3), the average relative magnetic permeability on each red point can be calculated as Equation (8). Symbols i and j are used to represent the point location, e.g., µ_11,x_ represents the horizontally measured relative magnetic permeability at the point on the top left corner.

(8) $$\mu_{\mathrm{ij},\mathrm{ave}}=\frac{\mu_{\mathrm{ij},x}+\mu_{\mathrm{ij},y}}{2}$$

### 3.5. Plate Test Results

#### 3.5.1. Fibre Spatial Distribution

Detailed mean and standard deviation (STD) of relative magnetic permeabilities for all plates are listed in Table 5. It can be seen that with the increase of fibre content, the relative magnetic permeability also increased. Due to the limited testing depth, the magnetic permeability only increased with the increase of plate thickness from 15 to 35 mm but no obvious difference between thicknesses from 35 to 50 mm.

**Figure 8:**
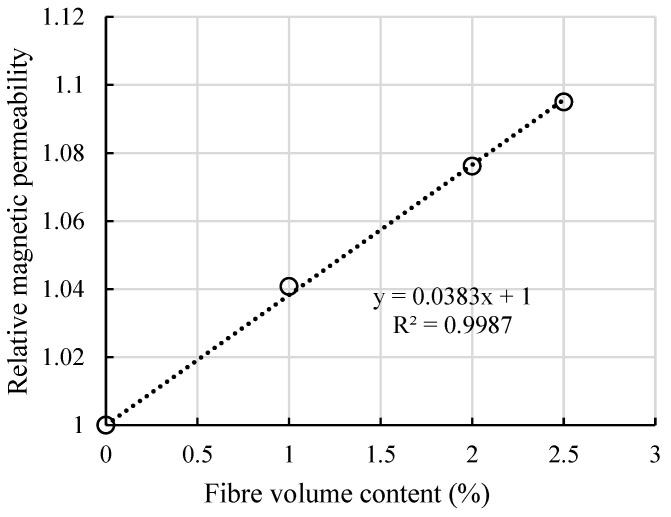
Relationship between fibre volume content and relative magnetic permeability.

Combining the results from Table 5 and the results from Nunes et al. [22], a relationship between fibre volume content and theoretical relative magnetic permeability µ can be derived as shown in Figure 8. When fibre volume content is 0, the relative magnetic permeability value equals to 1, which represents the magnetic permeability of air. The R-squared value of 0.9987 shows a nearly perfect linear fitting.

Considering the effective depth of the magnetic probe, if relative magnetic permeability µ_test_ was obtained on a thin specimen, the attenuation factor should be applied to calculate the real fibre volume content. The relationship between real relative magnetic permeability µ_r_ and fibre volume content V_f_ is further developed into

(9) $$\mu_{r}=\frac{\mu_{\mathrm{test}}-1}{1-{\mathrm{AF}}_{t}}+1$$

(10) $$\mu_{r}=0.0383\;\times {\;V}_{f}+1$$

where AF_t_ represents the attenuation factor when the specimen’s thickness is t. Combining Equations (9) and (10), the corrected relationship can be expressed as:

(11) $$V_{f}=\frac{\mu_{\mathrm{test}}-1}{0.0383\times \left(1-{\mathrm{AF}}_{t}\right)},$$

By using Equation (11), real fibre volume contents can be derived and are indicated in Table 6. For all the 1% vol. plates, the fibre content fulfilled the designed requirement. For 2% and 2.5% vol., some plates possessed lower fibre volume content than the designed value. 

With the increase of fibre volume content, the standard deviation also increased, which revealed that fibre tended to distribute more non-uniformly. This effect was more severe with the 2.5% vol. UHPFRC plates. A possible reason was the fibre balling or gathering effect. Although there were variations within each plate, the differences were very small compared to the total fibre volume content. Therefore, the plate can be considered almost uniformly spatially distributed.

**Figure 9:**
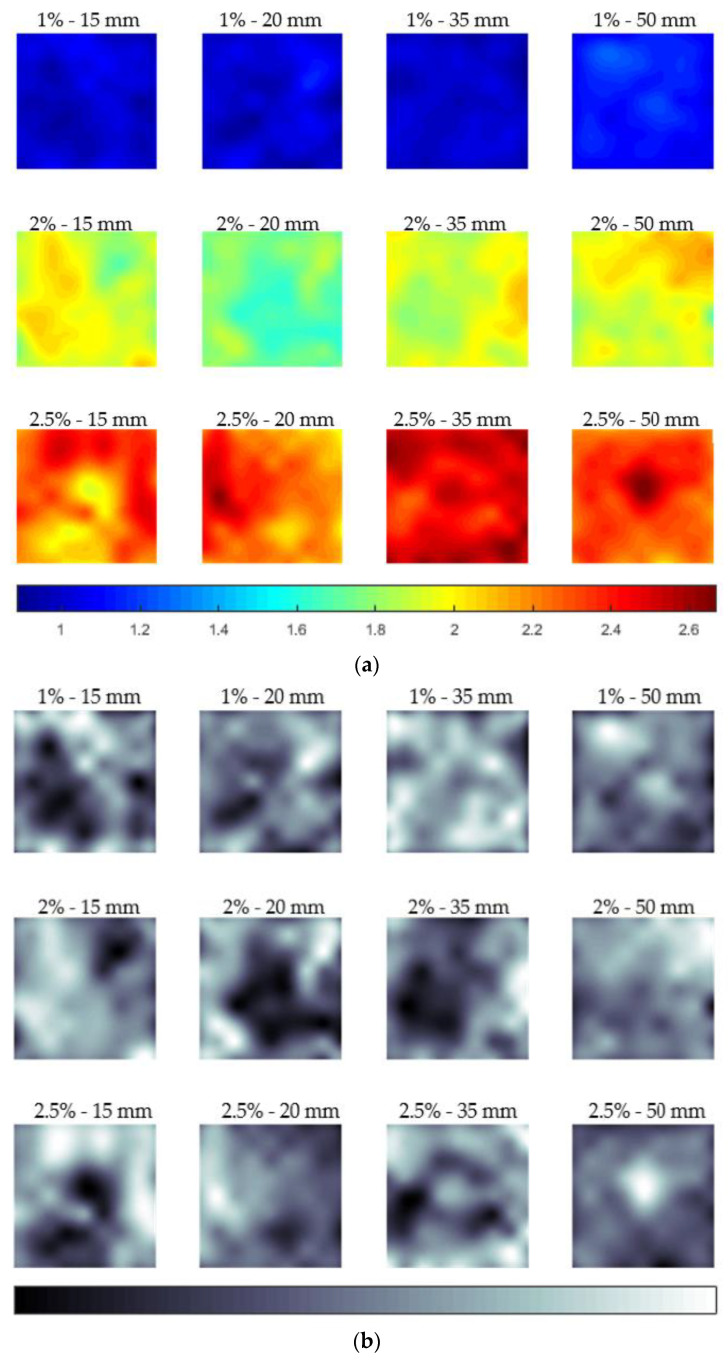
Fibre volume content distribution of all plates. (**a**) With a scale as the fibre volume content in percentage and (**b**) with a general non-unified scale.

The first coloured contour plot in Figure 9a describes the fibre distribution of all plates at a unified scale. Colours ranging from blue to red represent the differences of fibre volume content. It can be seen directly that plates 2%–20 mm and 2.5%–15 mm have a lower fibre volume content than the designed fibre volume content. The detailed fibre distribution cannot be visualized, since the range of data was too wide in the coloured contour plots. Thus, a greyscale contour plot is given in Figure 9b as a comparison. The darker shading indicates a lower fibre volume content. It can be seen that there was no obvious fibre spatial distribution trend in the middle area of each plate, only the four boundaries appeared to have a lower fibre volume content. This mainly results from the limitation of the testing area (boundary effect).

#### 3.5.2. Fibre Orientation Distribution

Based on previous literature by Nunes et al. [22], the fibre orientation was expressed by an orientation indicator ρ_Δ_. The orientation indicator of the red points can be calculated as:

(12) $$\rho_{\Delta }=\frac{\mu_{\mathrm{ij},y}-\mu_{\mathrm{ij},x}}{2\left(\mu_{\mathrm{ij},\;\mathrm{ave}}-1\right)}$$

Nunes et al. [22] found the orientation indicator had a sinusoidal relationship with the fibre orientation angle, but an analytical expression between the fibre orientation angle and orientation indicator ρ_Δ_ was not presented. Through further derivation, it was found that the orientation indicator is a function of polynomial terms of cos(φ). For example, the full expression of orientation indicator 1% vol. UHPFRC in terms of the orientation angle φ according to Nunes et al. [22] can be expressed as:

(13) $$\rho_{\Delta ,1\%}=\frac{-1076.68{\;\cos}^{6}\left(\varphi \right)+1615.03{\;\cos}^{4}\left(\varphi \right)+4.03\times 10^{7}{\;\cos}^{2}\left(\varphi \right)-2.02\times 10^{7}}{\begin{array}{c} (-0.96\;\cos^{6}\left(\varphi \right)+5.68\times 10^{5}\;\cos^{4}\left(\varphi \right)-5.68\times 10^{5}{\;\cos}^{2}\left(\varphi \right)\\ -6.41\times 10^{7}+0.48{\;\cos}^{2}\left(\varphi \right)) \end{array}}$$

After numerical analysis, only the constant terms and the cos^2^(φ) term on the numerator were found to be critical to the value of fibre orientation indicator ρ_Δ_. Therefore, Equation (13) can be further simplified to:

(14) $$\rho_{\Delta ,1\%}\approx -0.63\;\times \cos^{2}\left(\varphi \right)+0.315=0.315\times \;\cos\left(2\varphi \right)$$

**Figure 10:**
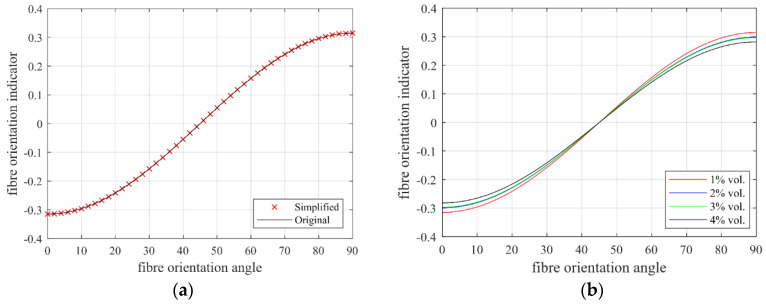
Relationship between fibre orientation angle (unit: degree) and fibre orientation indicator. (**a**) Comparison between simplified and original equation and (**b**) comparison between different fibre content.

Figure 10a shows the comparison between the original fibre orientation indicator calculated using Equation (13) and the simplified orientation indicator calculated using Equation (14). No obvious difference can be observed from 0 to 90°. Therefore, the original equation can be replaced with the simplified equation. There also works with other fibre volume percentages.

The relationship for other fibre percentage is approximately equal (≈) to:

(15) $$\rho_{\Delta ,2\%}\approx -0.299\times \;\cos\left(2\varphi \right)$$

(16) $$\rho_{\Delta ,3\%}\approx -0.297\;\times \cos\left(2\varphi \right)$$

(17) $$\rho_{\Delta ,4\%}\approx -0.282\;\times \cos\left(2\varphi \right)$$

This relationship was drawn in Figure 10b. It can be seen that the fibre volume content did not have a significant effect on the fibre orientation indicator, especially when the fibre orientation angle was around 45°. Generally, the fibre orientation indicator can be expressed as:

(18) $$\rho_{\Delta }=a\;\times \cos\left(2\varphi \right)$$

where the fibre orientation indicator coefficient slightly ranges around −0.3 depending on the fibre volume content.

**Figure 11:**
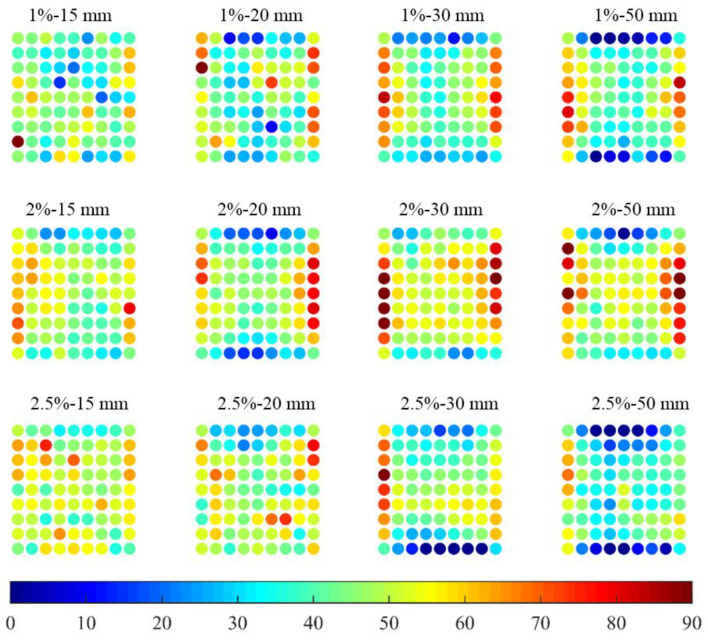
Fibre orientation angle distribution of all plates.

By using Equation (18), the fibre orientation distributions can be characterized. Figure 11 shows the fibre orientation distribution of all plates. Instead of contour plots, the fibre orientation angle is represented by dots in different colours. The fibre orientation ranged from 0 to 90° from the horizontal axis. It can be seen that fibres tended to orient at 0° at the top and bottom boundaries, while orienting at 90° along the left and right boundaries. There are two possible explanations: firstly, fibre tends to align their orientation to the mould due to the wall effect of the plate mould boundary; and secondly, fibre contents at the four boundaries were lower compared to other parts, which resulted from the testing method. The vertical testing value at the top and bottom points and the horizontal testing value at the furthest left and right points were lower.

**Figure 12:**
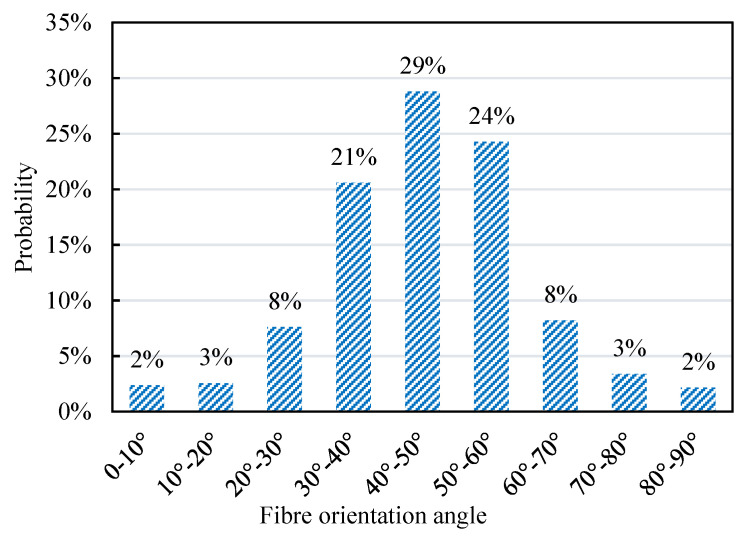
Distribution of fibre orientation angles.

Figure 12 shows the distribution of fibre orientation angle based on 972 (9 × 9 × 12) sample points. It can be seen from the graph that under this specific casting method, the distribution of the fibre orientation angle generally follows a normal distribution and most fibres orient at an angle between 40° and 50°. The normal distribution function was calculated based on the mean and standard deviation values of fibre orientation angle, which can be represented as:

(19) $$f\left(\varphi \right)\;=\frac{1}{\sqrt{2\pi }\sigma }\exp\left(\frac{{\left(\varphi -\zeta \right)}^{2}}{2\sigma^{2}}\right)$$

where the mean value σ = 45.60° and standard deviation ζ = 15.32°.

Detailed mean and STD value of fibre orientation angles can be seen in Table 7. The increase of fibre content and fibre distribution do not have a direct relationship with the fibre orientation angle. Due to the wall effect, the coefficient of variance for the fibre orientation angle was higher comparing to the fibre volume content.

## 4. Mechanical Test Results

### 4.1. Compressive Test

The compressive test was carried out on 0, 1%, 2%, and 2.5% specimens 5 days after curing. Six specimens were tested from each group. With the increase of fibre content, the compressive strength steadily increased in an almost linear trend. Detailed compressive test results can be seen in Table 8.

### 4.2. Flexural Test

**Figure 13:**
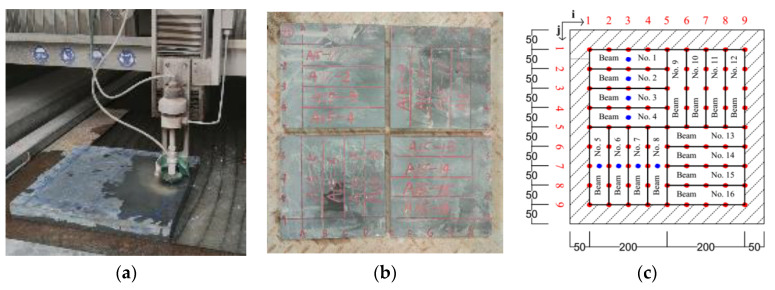
Concrete plate cutting. (**a**) Water blade cutting; (**b**) concrete plate after cutting; and (**c**) schematic graph.

All the 500 mm × 500 mm × 15 mm, 500 mm × 500 mm × 20 mm, 500 mm × 500 mm × 35 mm, and 500 mm × 500 mm × 50 mm plates were firstly cut into four sections by water blade to maintain the accuracy of dimension (Figure 13a,b). Then, the 250 mm × 250 mm × 15 mm, 250 mm × 250 mm × 20 mm, 250 mm × 50 mm × 35 mm, and 250 mm × 250 mm × 50 mm plates were further cut into 200 mm × 50 mm × 15 mm, 200 mm × 50 mm × 20 mm, 200 mm × 50 mm × 35 mm, and 200 mm × 50 mm × 50 mm. The detailed cutting scheme can be seen in Figure 13c. Beam No. 1–8 were tested in this research.

**Figure 14:**
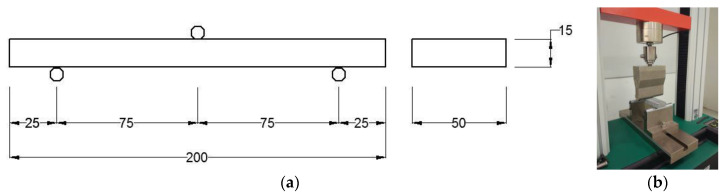
Three-point bending test for the UHPFRC beam. (**a**) Schematic graph (unit: mm) and (**b**) experimental set-up.

The flexural test were conducted 5–7 days after curing. The experiment was carried out on a 3-ton universal testing machine at a constant deflection control speed of 0.3 mm/min. Taking a 15 mm thick beam as an example, the three-point bending test setup can be seen in Figure 14. The effective span was 150 mm. The experiment was terminated once the load dropped below 50% of the peak load. The displacement movement of the machine was used to plot the load–displacement diagram

The flexural strength for a three-point bending test can be calculated from Equations (20) to (22).

(20) $$M=\frac{F_{f}\times L}{4}$$

(21) $$W=\frac{{\mathrm{BH}}^{2}}{6}$$

(22) $$f_{f}=\frac{M}{W}$$

where

F_f_  peak flexural load;
L  distance between support and the nearby loading point;
M  external moment;
W  section modulus;
B  width of the beam cross section;
H  height of the beam cross section;
f_f_  flexural strength.

Average first crack flexural strength and peak flexural strength of eight beams of each plate can be seen in Table 9. The merged cell on the right side shows the average value of four plates. For UHPC, no micro or macrocracks were observed before reaching peak load. The first crack strength equalled the peak flexural strength. 

## 5. Correlation between Magnetic Probe Test and Mechanical Test

To correlate the results between the fibre distribution test and the mechanical test for each beam, the relative inductance values of the blue points in Figure 13c are also needed. The value is derived from the nearby two red points. For the blue points of Beams 1–4, the average relative magnetic permeability can be expressed as:

(23) $$\mu_{\mathrm{ij},\;\mathrm{ave}}=\frac{\mu_{\mathrm{ij},x}+\mu_{\mathrm{ij},y}+\mu_{i\left(j+1\right),x}+\mu_{i\left(j+1\right),y}}{4}$$

For Beams 5–8, it can be calculated by:

(24) $$\mu_{\mathrm{ij},\;\mathrm{ave}}=\frac{\mu_{\mathrm{ij},x}+\mu_{\mathrm{ij},y}+\mu_{\left(i+1\right)j,x}+\mu_{\left(i+1\right)j,y}}{4}$$

For the red points in Figure 13c, the fibre orientation indicator can be calculated straightly using Equation (12). For the blue points, the orientation indicator can be derived from the nearby two red points. For the blue points on Beams 1–4, the orientation indicator ρ_Δ_ can be derived as:

(25) $$\rho_{\Delta }=\frac{\mu_{\mathrm{ij},y}+\mu_{i\left(j+1\right),y}-\mu_{\mathrm{ij},x}-\mu_{i\left(j+1\right),x}}{\mu_{\mathrm{ij},y}+\mu_{i\left(j+1\right),y}+\mu_{\mathrm{ij},x}+\mu_{i\left(j+1\right),x}-4}$$

(26) $$\rho_{\Delta }=\frac{\mu_{\mathrm{ij},y}+\mu_{\left(i+1\right)j,y}-\mu_{\mathrm{ij},x}-\mu_{\left(i+1\right)j,x}}{\mu_{\mathrm{ij},y}+\mu_{\left(i+1\right)j,y}+\mu_{\mathrm{ij},x}+\mu_{\left(i+1\right)j,x}-4}$$

**Figure 15:**
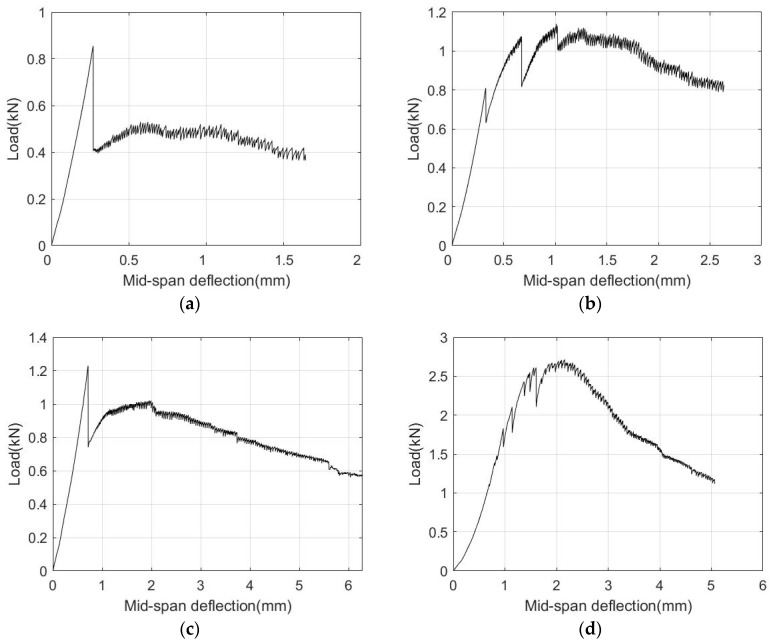
Load–deflection curves for 200 mm × 50 mm × 20 mm UHPFRC beams. (**a**) 1%-15-s6; (**b**) 1%-15-s4; (**c**) 2%-20-s8; and (**d**) 2%-20-s5.

Figure 15 shows four typical load-deflection curves for 1% and 2% vol. UHPFRC. Both load-softening and load-hardening behaviours can be observed. Statistically, only 9 out of 32 1% vol. UHPFRC beams had obvious strain hardening behaviour. For 2% vol. UHPFRC, 27 out of 32 beams had load-hardening behaviour after the first crack. For 2.5% vol. UHPFRC beams, 29 out of 31 beams had load-hardening performance.

**Figure 16:**
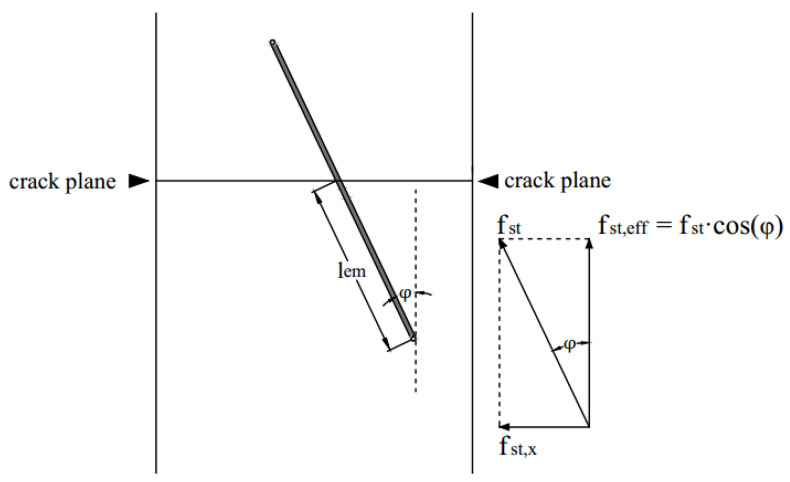
Effective embedment length of fibres inside the concrete matrix in the uniaxial tensile test in fibre activation stage.

Apart from the tensile property of the UHPC matrix, both the fibre orientation and fibre volume content can determine whether the beam performs a load-hardening behaviour or not. Initially in the uniaxial tensile test, fibres were fully bonded, and the tensile load was mainly carried by the concrete matrix. Thus, the first crack tensile strength mainly depended on the tensile properties of the concrete matrix, which agrees with previous researchers [28,29]. After the concrete cracks, concrete hardly sustained any loads, but fibres were not pulled-out yet due to the static frictional force τ between fibres and the concrete matrix. With the increase of uniaxial tensile load, the static frictional force also increased. Whether the beam had a load-hardening or load-softening performance depends on whether the whole fibre–concrete interfaces can provide enough frictional force, while the frictional force is related to the roughness of the interface, number of fibres (fibre volume content), and effective embedment length (fibre orientation). As can be seen in Figure 16, the effective static frictional force f_st,eff_ carried by one fibre can be estimated as:

(27) $$f_{\mathrm{st}}=\pi \times d_{f}\times l_{\mathrm{em}}\times \tau$$

(28) $$f_{\mathrm{st},\mathrm{eff}}=f_{\mathrm{st}}\times \cos\varphi =\pi \times d_{f}\times l_{\mathrm{em}}\times \tau \times \cos\varphi$$

where

f_st_  static frictional force;
f_st,eff_  effective static frictional force;
d_f_  fibre diameter;
l_em_  effective embedment length;
τ  static frictional strength;
φ  fibre orientation angle.

The total effective static frictional force f_st,n_ can be calculated as:

(29) $$f_{\mathrm{st},n}=f_{\mathrm{st},\mathrm{eff}}\times n_{f}=\pi \times d_{f}\times l_{\mathrm{em}}\times \tau \times \cos\varphi \times n_{f}$$

**Figure 17:**
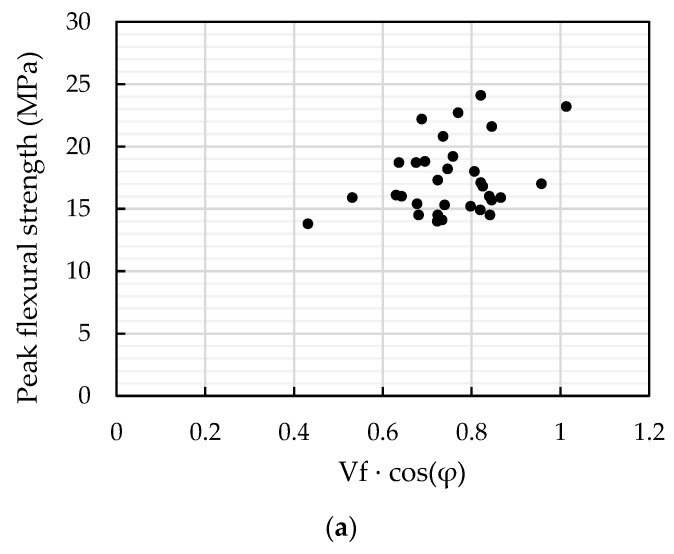

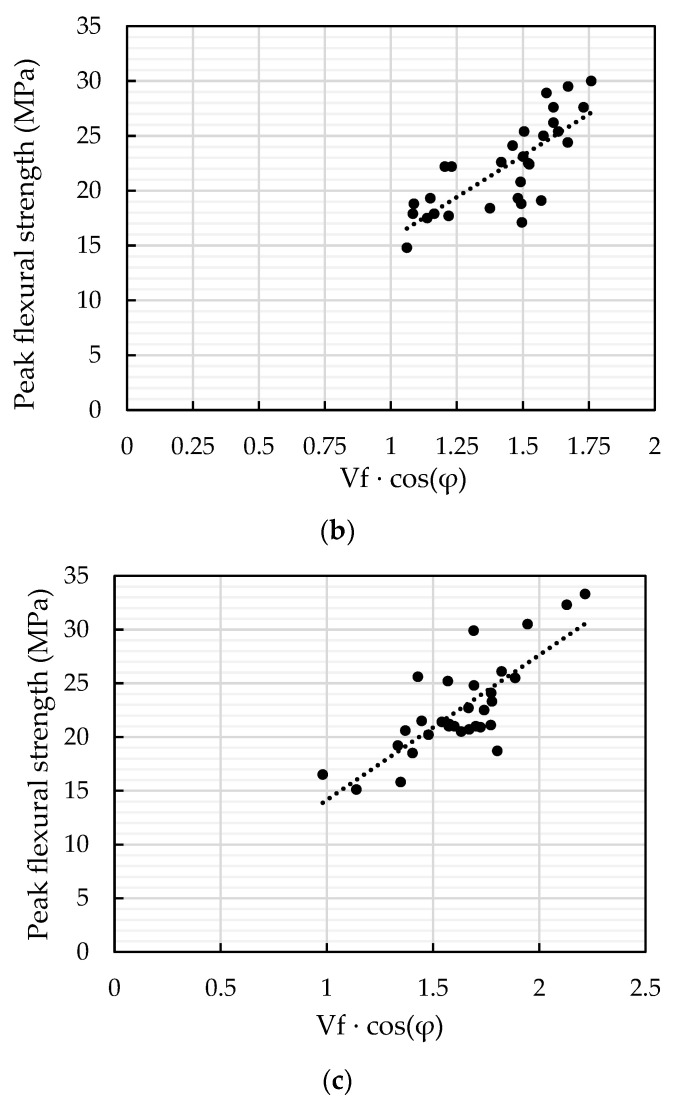
Relationship between V_f_ × cos(φ) and peak flexural strength. (**a**) 1% vol. UHPFRC; (**b**) 2% vol. UHPFRC; and (**c**) 2.5% vol. UHPFRC.

Assuming the static frictional strength is constant in Equation (29), it can be seen that the total effective static frictional force has a linear relationship with V_f_ cos(φ) and number of fibres across the crack plane. Figure 17 shows the relationship between V_f_ cos(φ) and the peak flexural strength. For 1% UHPFRC, there is no obvious trend, mainly owing to the insufficient frictional force to support load-hardening performance, therefore, the peak flexural strength was determined by the properties of the concrete matrix. A linear relationship can be found for 2% and 2.5% UHPFRC. If the concrete matrix w consistent, the relationship between the uniaxial tensile strength f_t,φ1_, f_t,φ2_ at different fibre orientation angles φ_1_, φ_2_ can be calculated as:

(30) $$\frac{f_{t,\varphi 1}}{\cos\left(\varphi_{1}\right)\times V_{f1}}=\frac{f_{t,\varphi 2}}{\cos\left(\varphi_{2}\right)\times V_{f2}}$$

## 6. Conclusions

This research focused on investigating fibre distribution of UHPFRC using C-shape magnetic probe. The following conclusions can be drawn.

- The effective testing depth of the C-shape magnetic probe was firstly determined by curve fitting analysis using MATLAB. An exponential equation was derived to alter the relative magnetic permeability value, and to correlate with the real fibre volume content for elements with different thicknesses.
- The magnetic permeability appeared to be linearly correlated with fibre volume content. With the increase of fibre volume content, the standard deviation of magnetic permeability also increased, which revealed a more non-uniform distribution of fibres.
- The analytical solution for deriving the fibre orientation indicator based on fibre orientation angle was derived. A fibre orientation indicator coefficient *a* was determined to be around −0.3.
- With a low fibre amount or large fibre orientation angle, the peak flexural strength and uniaxial tensile strength were more related to the tensile property of the concrete matrix. No strain hardening behaviour occurred after concrete cracks.
- With a high fibre amount or a small fibre orientation angle, the peak flexural strength and uniaxial tensile strength were closely affected by the static frictional force between the concrete matrix and fibres. Thus, strain hardening behaviour occurred. Results show that the uniaxial tensile strength had a linear relationship with the V_f_ cos(φ) value.

## Figures and Tables

**Table 1:** Mix proportion for pre-mix ultra-high performance fibre reinforced concrete (UHPFRC; provided by third party company).

Material	Content (kg/m^3^)
Dry-Mix	2200
Water	192
Steel Fibre	0, 78, 156, 195 (for 0%, 1%, 2%, and 2.5% vol.)
Superplasticizer	20.5
Total	2413, 2491, 2569, 2608 (for 0%, 1%, 2%, and 2.5% vol.)

**Table 2:** Specimen list and casting purpose for different types of specimens.

Shape	Dimension (mm)	Casting Purpose
Cube	100 × 100 × 100	Compressive test
Plate	500 × 500 × 15	Flexural test, Magnetic probe test
Plate	500 × 500 × 20	Flexural test, Magnetic probe test
Plate	500 × 500 × 35	Flexural test, Magnetic probe test
Plate	500 × 500 × 50	Flexural test, Magnetic probe test

**Table 3:** Mixing procedure and time for premix UHPFRC.

Procedure	Duration (min)
Mix Dry-Mix Material	1
Add Superplasticizer and 2/3 Water	6
Add 1/3 Water	3
Add Fibres	1

**Table 4:** Average attenuation factors calculated for each group.

t (mm)	2% vol.—A	2% vol.—B	2.5% vol.—A	2.5% vol.—B
0	100%	100%	100%	100%
2	75%	72%	77%	73%
4	57%	54%	57%	58%
8	40%	39%	39%	40%
12	23%	23%	24%	26%
15	19%	19%	16%	19%
17	14%	15%	13%	15%
20	10%	13%	12%	12%
24	6%	7%	7%	6%
32	3%	2%	4%	4%
36	3%	3%	3%	3%

**Table 5:** Mean and standard deviation (STD) of relative magnetic permeabilities of all plates.

Index	Fibre Volume Content	15 mm	20 mm	35 mm	50 mm
Mean	1%	1.031	1.035	1.038	1.043
	2%	1.059	1.060	1.074	1.075
	2.5%	1.069	1.078	1.095	1.088
Standard Deviation	1%	0.0004	0.0005	0.0002	0.0005
	2%	0.0005	0.0006	0.0006	0.0012
	2.5%	0.0016	0.0009	0.0008	0.0017

**Table 6:** Mean and STD of fibre volume content of all plates.

Index	Fibre Volume Content	15 mm	20 mm	35 mm	50 mm
Mean	1%	1.0%	1.0%	1.0%	1.1%
	2%	1.9%	1.7%	1.9%	2.0%
	2.5%	2.2%	2.2%	2.5%	2.3%
Standard Deviation	1%	0.013%	0.016%	0.006%	0.014%
	2%	0.018%	0.018%	0.016%	0.030%
	2.5%	0.052%	0.026%	0.020%	0.045%

**Table 7:** Mean and STD of fibre orientation angles of all plates.

Index	Fibre Volume Content	15 mm	20 mm	35 mm	50 mm
Mean	1%	43	43	45	41
	2%	48	46	54	51
	2.5%	51	48	43	35
Standard Deviation	1%	2.8	3.1	3.0	2.6
	2%	2.2	2.4	4.0	3.0
	2.5%	2.0	1.2	3.3	3.7

**Table 8:** Compressive strength of different groups of 100 mm cube specimens.

Fibre Volume Content	Average	STD	COV
0	87.8	4.7	5.4%
1%	134.1	5.3	4.0%
2%	141.4	4.4	3.1%
2.5%	149.5	5.4	3.6%

**Table 9:** First crack flexural strength and peak flexural strength (unit: MPa).

Fibre Volume Content—Thickness	First Crack Flexural Strength		Peak Flexural Strength	
0–15	20.0	18.5	20.0	18.5
0–20	18.6		18.6	
0–35	15.0		15.0	
0–50	20.3		20.3	
1%–15	15.1	16.3	16.0	17.4
1%–20	13.9		16.0	
1%–35	20.2		21.0	
1%–50	15.8		16.6	
2%–15	18.5	18.6	22.4	22.6
2%–20	16.8		21.4	
2%–35	19.6		25.0	
2%–50	19.4		21.8	
2.5%–15	18.0	17.9	22.2	22.6
2.5%–20	16.2		20.9	
2.5%–35	18.3		24.1	
2.5%–50	18.9		23.1	
